# Protective efficacy of Zika vaccine in AG129 mouse model

**DOI:** 10.1038/srep46375

**Published:** 2017-04-12

**Authors:** K. Sumathy, Bharathi Kulkarni, Ravi Kumar Gondu, Sampath Kumar Ponnuru, Nagaraju Bonguram, Rakesh Eligeti, Sindhuja Gadiyaram, Usha Praturi, Bhushan Chougule, Latha Karunakaran, Krishna M. Ella

**Affiliations:** 1 R&D Department Bharat Biotech International Ltd. Genome Valley, Shamirpet Hyderabad – 500 078, India

## Abstract

Zika virus (ZIKV) is a mosquito-borne flavivirus that causes asymptomatic infection or presents only mild symptoms in majority of those infected. However, vaccination for ZIKV is a public health priority due to serious congenital and neuropathological abnormalities observed as a sequelae of the virus infection in the recent epidemics. We have developed an inactivated virus vaccine with the African MR 766 strain. Here we show that two doses of the vaccine provided 100% efficacy against mortality and disease following challenge with homotypic MR 766 and the heterotypic FSS 13025 ZIKV strains in the Type I and Type II interferon deficient AG129 mice. Two doses of the vaccine elicited high titer of neutralizing antibodies in Balb/c mice, and the vaccine antisera conferred protection against virus challenge in passively immunized mice. The studies were useful to rationalize vaccine doses for protective efficacy. Furthermore, the vaccine antisera neutralized the homotypic and heterotypic ZIKV strains *in vitro* with equivalent efficiency. Our study suggests a single ZIKV serotype, and that the development of an effective vaccine may not be limited by the choice of virus strain.

Zika virus (ZIKV) targets neural progenitor cells during infection in pregnancy and causes fetal growth restriction, microcephaly and other congenital neurological abnormalities in humans^1^,^2^,^3^ and mice^4^,^5^. The virus infection causes Guillain-Barré syndrome (GBS) with acute inflammatory demyelinating neuropathy^6^,^7^. Higher incidence of GBS was observed during the virus epidemic in several countries^8^,^9^,^10^. The development of a safe and effective prophylactic vaccine is therefore a public health priority in countries with susceptible mosquito vectors and a large ZIKV naïve population. Dynamic disease modelling based on international travel, climatic conditions and the occurrence of competent *Aedes* mosquitoes predict new areas in Africa and Asia with a large population at risk from potential ZIKV infection^11^.

Several platform technologies for ZIKV vaccines are in development^12^,^13^,^14^. An inactivated virus vaccine elicited ZIKV envelope specific neutralizing antibodies and protected non-human primates (NHP) against challenge with the virus strains from Brazil and Puerto Rico^15^. A single dose of recombinant Rhesus adenovirus serotype 52 vector vaccine, or plasmid DNA vaccines expressing pre-membrane and envelope (prME) proteins elicited neutralizing antibodies and protected monkeys against viremia after virus challenge^15^,^16^,^17^. The non-human primate model is useful to derive correlates of protection for vaccine studies, but does not re-capitulate all the clinical signs observed in humans. The AG129 mouse model supports efficient ZIKV replication with high viral loads in organs, and exhibits severe disease symptoms with progression to mortality^18^. The AG129 mice lack both IFN−α/β and −γ receptors, but elicit B-cell and T-cell responses to infection^19^. As Type I interferon signalling in B-cells and CD4^+^ T-cells is required for optimal antibody response^20^, vaccine studies in this mouse model do not provide a full measure of immune correlates of protection. Nevertheless, it is an effective animal model to study vaccine efficacy against viremia, disease pathogenesis and mortality. The AG129 mouse model was used to test the efficacy of Dengue^21^ and Chikungunya vaccines^22^. Inactivated virus vaccines generally have a good safety profile, and in the case of ZIKV, is the preferred platform technology for deployment in an emergency epidemic situation where women of childbearing age would be the prime target population for vaccination. Here we show that the purified inactivated ZIKV vaccine developed using the MR 766 strain protected against viremia and clinical disease in AG129 mice. However, the choice of vaccine strain required demonstration of equivalent protective efficacy against the homologous and heterologous strains, as MR 766 belongs to the East African genotype, while the recent ZIKV epidemics are caused by the Asian genotype. The MR 766 strain was isolated in 1947 from a sentinel monkey in Uganda^23^. The FSS 13025 strain used in virus challenge studies was isolated in 2010 from Cambodia^24^ and belongs to the Asian genotype. Protective efficacy in AG129 mice complemented the robust immune response elicited by the vaccine in Balb/c mice.

## Results

### Vaccine efficacy in AG129 mice

**Figure 1:**
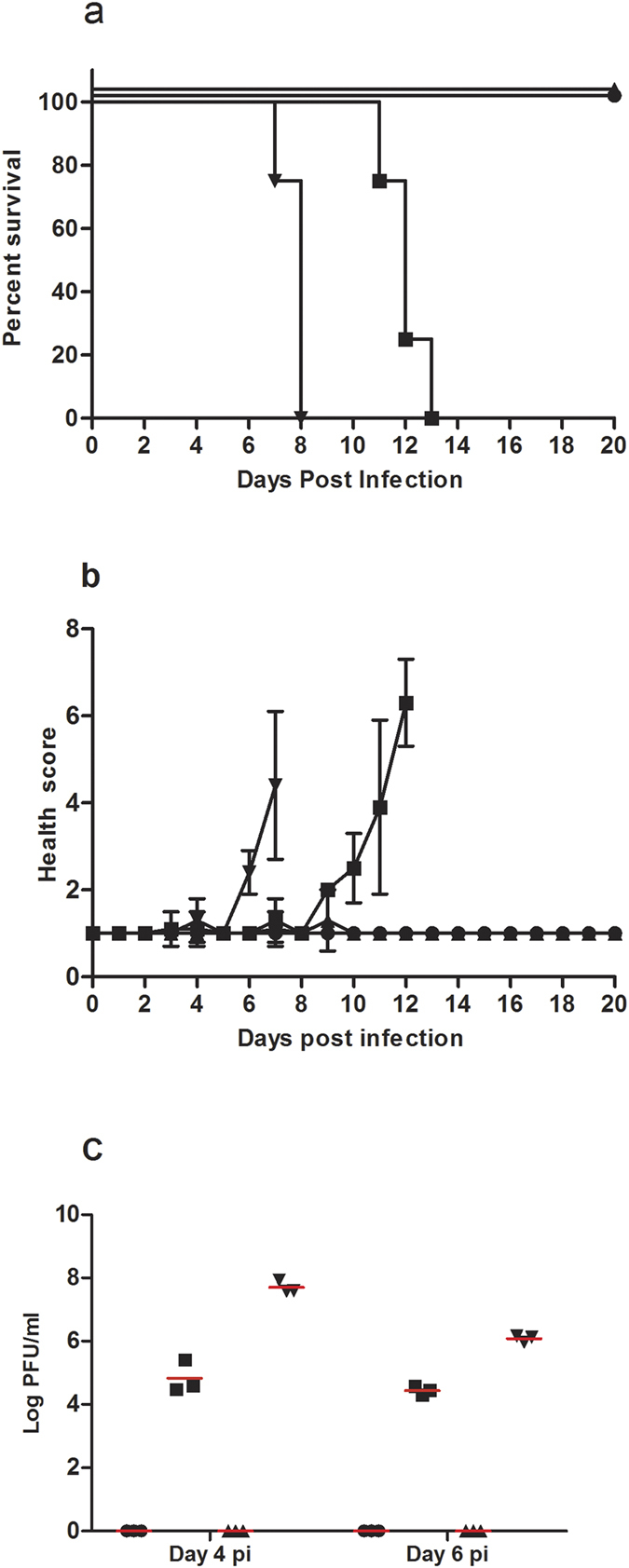
(**a**–**c**) Protective efficacy of ZIKV vaccine in AG129 mice. (**a**) Percent survival in vaccinated AG129 mice after challenge with 10^4^ PFU of (●) ZIKV-FSS 13025, or (▲) ZIKV-MR 766, and in the control mice that received equivalent titers of (■) ZIKV-FSS 13025, or (▼) ZIKV-MR 766. The mice in all four groups (*n* = 8/group) were monitored for survival up to 20 days after virus challenge. (**b**) Mean health scores of the four groups following virus challenge are depicted for 20 days. Health scores were calculated according to the parameters described in Table 1. Error bars represent s.d. (**c**) Viremia was estimated on days 4 and 6 after virus challenge in three animals from each group, and expressed as PFU/ml. Red bars reflect the mean of three values. The results are shown from a single experiment.

Two groups of 4–6 week old female AG129 mice (*n* = 8/group) were vaccinated with 10 μg per dose of the alum adsorbed purified inactivated vaccine on days 0 and 21 by intramuscular route, and challenged subcutaneously with 10^4^ PFU (plaque forming unit) of FSS 13025 or MR 766 on day 28 as schematically outlined in Supplementary Fig. S1a,b. Two groups of control mice (*n* = 8/group) received equivalent concentration of alum (placebo) and were challenged with 10^4^ PFU of FSS 13025 or MR 766. All the four groups were monitored daily for 20 days post infection for clinical health, mortality, body weight and temperature. Vaccinated mice were fully protected (100% survival) against infection with either of the virus strains. Death in the control groups challenged with MR 766 occurred earlier with mean time to death (MTD) of 8 days as compared to the group challenged with FSS 13025 with MTD of 12 days (Fig. 1a). Mean health scores for clinical disease based on appearance, mobility and alertness were calculated based on observations described in Table 1. The vaccinated group had a perfect health score of 1 throughout the observation period and no disease symptoms were observed, whereas the control animals exhibited progressive morbidity before succumbing to infection (Fig. 1b). Clinical score was supported by the lack of weight loss in the vaccinated animals (Supplementary Fig. S2a,b). Serum viremia was estimated on days 4 and 6 post infection in 3 animals from each group. Vaccinated mice exhibited undetectable viral load, whereas viremia peaked on day 4 in the control animals regardless of the challenge virus (Fig. 1c). Disease progression was more rapid in MR 766 group when compared to the FSS 13025 group. Body temperatures across all the groups did not vary greatly with the exception of the control MR 766 group which had a large temperature drop just prior to death (Supplementary Fig. S3a,b). The vaccine protected against mortality and disease caused by ZIKV strains of the Asian and African genotypes in the AG129 mice.

### Immunogenicity

**Figure 2:**
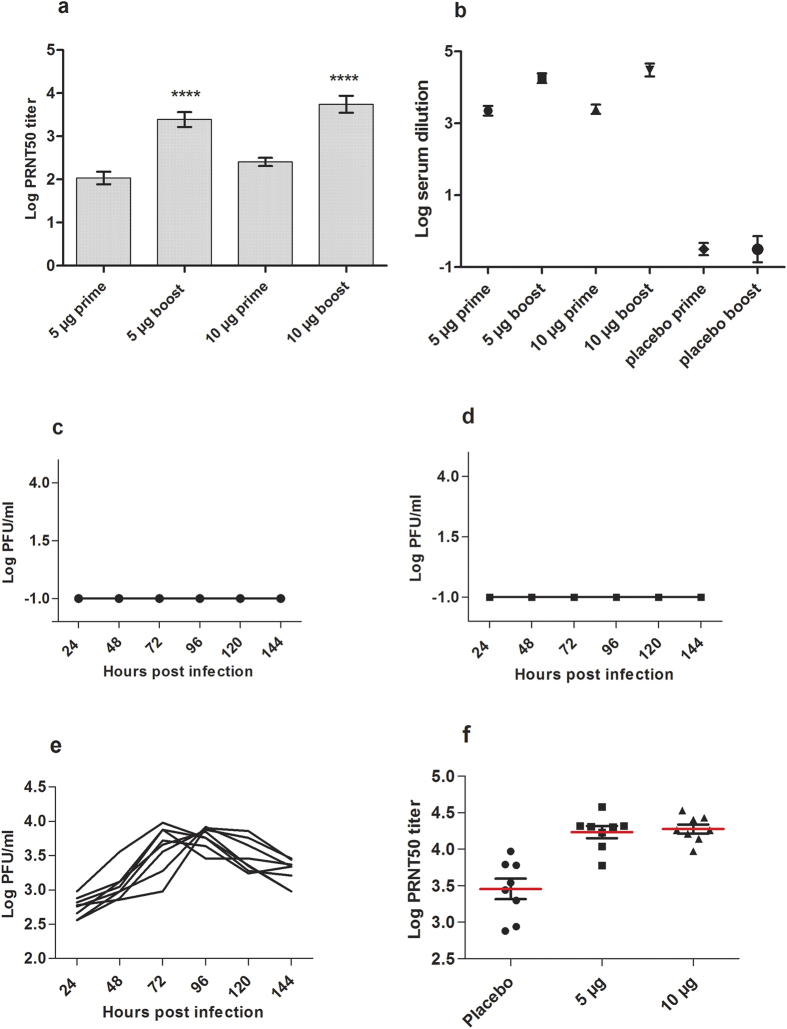
(**a**–**f**) Immunogenicity of Zika vaccine. (**a**) Serum neutralizing antibody titers by PRNT_50_ following vaccination with two doses of 5 μg or 10 μg per dose in Balb/c mice (*n* = 8/group) is depicted as mean log titers with 95% c.i. and (**b**) antibody binding to purified inactivated ZIKV was estimated by ELISA and the antibody titers are expressed as mean log serum dilution with 95% c.i. The increase in antibody titers by PRNT_50_ and ELISA were significant (*P* < 0.0001) when compared by two-tailed paired t-test between the prime and boost in both the dose groups. Unpaired t-test between the 5 μg and 10 μg prime, and between 5 μg and 10 μg boost doses gave *P* values of 0.64 and 0.031 respectively in two-tailed analyses. (**c**) Viremia in each of the vaccinated mice up to 144 hours after challenge with 10^5^ PFU of ZIKV MR 766 in the 5 μg and in (**d**) the 10 μg dose groups was undetectable. (**e**) Plasma virus titers in the individual placebo control recipient mice up to 144 hours after virus challenge. (**f**) Serum neutralizing antibody titers 7 days after virus challenge in the placebo control, and in the 5 and 10 μg vaccinated groups are expressed as mean log PRNT_50_ titers. The red bar is the mean and the error bars represent 95% c.i. by One-way analysis of variance (ANOVA). All the samples at each time point were assayed in triplicates, and the results are from a single experiment.

To study the immune responses induced by the alum adsorbed purified inactivated ZIKV vaccine, we immunized Balb/c mice (*n* = 8/group) with two doses of 5 μg or 10 μg of the vaccine, and the control group with alum (placebo) by intramuscular route on day 0 and on day 21 as schematically outlined in Supplementary Fig. S1a,b. Vaccinated mice developed ZIKV neutralizing antibodies on day 14 after prime dose, and on day 28, one week after boost immunization. Mean log neutralizing antibody titers by PRNT_50_ were 2.14 after prime, and 3.38 after boost dose in the 5 μg group (*P* < 0.0001), and 2.41 and 3.74 in the 10 μg group (*P* < 0.0001) (Fig. 2a). The control animals did not develop detectable antibody responses and hence the results are not shown. The corresponding mean log antibody titers by ELISA using purified inactivated ZIKV as the coating antigen were 3.35 and 4.26 (*P* < 0.0001) in the 5 μg group, and 3.50 and 4.41 (*P* < 0.0001) in the 10 μg group (Fig. 2b). Protective efficacy of the vaccine was assessed in the vaccinated and in control groups by intravenous injection of 10^5^ PFU of MR 766 and the virus titers in plasma were estimated every 24 hours up to 144 hours post-infection. Viremia was undetectable in the 5 μg and 10 μg vaccine groups up to 144 hours (Fig. 2c,d), but peaked at 72–96 hours post-infection in the control animals (Fig. 2e). The vaccine protected against virus replication as infectious virus particles could not be detected in plasma by plaque assay even after three serial amplifications *in vitro* in Vero cells. Vaccinated animals showed good anamnestic response to virus challenge with saturating mean log PRNT_50_ titers of 4.30 and 4.26 in the 5 μg and 10 μg dose groups respectively (Fig. 2f).

### Passive immunization

**Figure 3:**
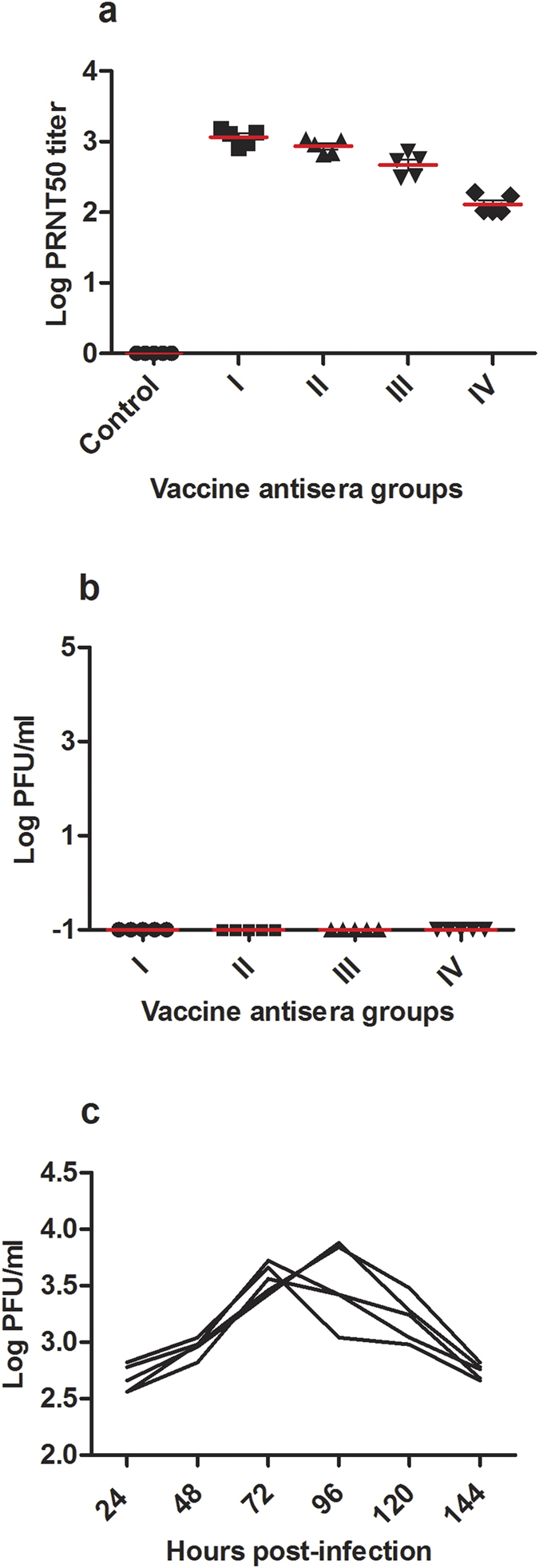
(**a**–**c**) Passive immunization in mice. (**a**) Serum neutralizing antibody titers in vaccine antisera recipient Balb/c mice (*n* = 5/group), 24 hours following intraperitoneal injection of two-fold serial dilutions of 1:1, 1:2, 1:4 or 1:8 of rabbit vaccine antisera (groups I, II, III and IV) and in rabbit pre-immune sera recipient control mice. (**b**) Undetectable plasma virus titers in passively immunized mice following challenge with 10^5.5^ PFU of ZIKV MR 766 by i.v. (**c**) Plasma virus titers in the control animals following virus challenge, expressed as PFU/ml. Red bars indicate mean values. All the samples from individual mice at each time point were assayed in triplicates, and the results are from a single experiment.

We tested the protective efficacy of the vaccine antisera to virus challenge by passive immunization in Balb/c mice. Rabbit vaccine antisera (log PRNT_50_ titer of 4.28) at either 1:1, 1:2, 1:4 or 1:8 dilutions (groups I–IV) was administered in a volume of 0.3 ml by i.p. route in Balb/c mice (*n* = 5/group). The animals were challenged 6 hours later with10^5.5^ PFU of MR 766 by i.v. The control animals received pre-immune serum by i.p. and equivalent virus dose by i.v. as the passively immunized animals. The serum mean log PRNT_50_ titers 24 hours after antisera administration in the recipient mice groups I to IV were 3.07, 3.02, 2.52, and 2.11 respectively (Fig. 3a). No infectious particles could be detected in animals from 24 to 144 hours after passive immunization (Fig. 3b), whereas viremia peaked at 72-96 hours in control animals that received pre-immune serum (Fig. 3c). The lack of detectable virus particles in the passively immunized mice was further confirmed by plaque assay after three serial amplifications *in vitro* in Vero cells.

### Vaccine cross-neutralization

While MR 766 vaccine protected against the homotypic and heterotypic virus challenge in AG129 mice, we tested if the vaccine sera cross neutralized the African and Asian strains with equivalent efficiency. The vaccine sera cross neutralized MR 766 and FSS 13025 with mean log PRNT_50_ titers of 4.25 and 4.26 respectively (Supplementary Fig. S4a,b), that suggests a single serotype of ZIKV.

## Discussion

The envelope E protein is the target of ZIKV neutralizing antibodies^25^,^26^. The Asian lineage of the virus has maintained an amino acid identity of 99.4 to 100% in the E protein (Supplementary Fig. S5) suggesting that immune pressure has not been a dominant factor in virus evolution and fitness, and that any Asian ZIKV strain can confer protective immunity against other strains of this lineage^27^. The ZIKV Asian strain FSS 13025 shares 99.2 to 99.8% amino acid identity in the E protein with the contemporary strains of the same lineage, while MR 766 shares 97.03 to 97.62% amino acid identity with the Asian strains in the same region (Supplementary Fig. S5). ZIKV convalescent sera from the recent epidemics neutralized H/PF/2013 isolated from French Polynesia in 2013, Paraiba/2015 strain from Brazil, and the MR 766 with similar efficiency^28^, and Rhesus macaques infected with MR 766 were protected when challenged with the heterologous H/PF/2013 strain^29^. While the studies with convalescent sera identified a single serotype, we show that the ZIKV vaccine derived from the African strain induced cross-lineage protective efficacy against a strain of the Asian genotype in an appropriate animal model, and the vaccine antisera neutralized the homotypic and heterotypic strains *in vitro* with equivalent efficiency. Our studies also showed rapid progression in disease pathogenesis with MR 766 as compared to equivalent challenge with FSS 13025 in AG129 mice. Vaccines derived from single antigenic serotype have also been effective against other flaviviruses such as Yellow fever virus^30^, Tick-borne encephalitis^31^,^32^, and Japanese encephalitis^33^,^34^ viruses that exist as multiple genotypes.

Any ZIKV MR 766 sequence data needs to be interpreted with caution as multiple MR 766 sequences in GenBank show deletion of the signature ‘VNDT’ glycosylation site in the E protein. Sequence heterogeneity could perhaps be due to passage history in different labs subsequent to the extensive passage of the original MR 766 strain in mouse brain^35^. Here we show that the MR 766 strain from ATCC which is the original repository of the virus has the conserved glycosylation motif and identical number of amino acids as the contemporary Asian strains. The twice plaque purified virus strain sequenced in this study, matches the sequence available in GenBank under the accession numbers KX377335, HQ234498 and KU720415, and henceforth should represent the correct sequence of MR 766.

The candidate MR 766 vaccine conferred protective efficacy comparable to vaccines developed using the ZIKV strains of the Asian genotype. The log median neutralizing antibody titers of 3.66 induced by two 5 μg doses of the purified inactivated PRVABC59 ZIKV strain in rhesus macaques^15^ is comparable to log mean neutralizing antibody titers of 3.38 induced by two equivalent doses of the inactivated MR 766 vaccine in Balb/c mice in this study. The values are also comparable to log antibody titers of ~3.0 elicited by two 50 μg doses of prME DNA vaccine in Balb/c mice^17^, and to two doses of H/PF/2013 strain prME DNA vaccine in non-human primates^16^. A single dose of 10^11^ virus particles of rhesus adenovirus 52 vectored vaccine encoding prME also induced comparable neutralizing antibody titers in rhesus macaques^15^. In all the studies, the vaccine-induced antibodies conferred sterilizing immunity against virus challenge either in mice and/or non-human primates. Passive antibody transfer studies suggests a threshold of ~2.0 (log neutralizing antibody titers) for conferring protection against virus infection in these animal models^15^,^16^. The values are comparable to log mean PRNT_50_ titer of ~2.11 that conferred complete protection against virus challenge reported in this study.

The ZIKV vaccine induced neutralizing antibody titers are much higher than the threshold of 1:10 PRNT_50_ titer shown to be protective for other flavivirus vaccines^36^. A single boost dose significantly enhanced the neutralizing and binding antibody titers. To further rationalize the protective doses, we found that there was no significant difference between the 5 μg and 10 μg of vaccine in prime and prime-boost immunization. Also, the neutralizing antibody titers induced by a single 5 μg dose was comparable to the log PRNT_50_ titer of ~2.11 that conferred passive protection against virus replication. Protection at lower antibody levels and duration of protective immunity conferred by passive antibody transfer were not studied. Vaccination conferred complete protection against viremia and disease up to 14 and 20 days after virus challenge which is 35 and 48 days after prime dose vaccination in Balb/c and AG129 mice respectively. A strong anamnestic response to virus challenge with high increase in mean neutralizing antibody titers indicates that vaccine could potentially confer longer duration of protective immunity when evaluated at similar dose levels and by the same route in clinical trials.

Safety evaluation of the ZIKV vaccine in clinical trials have to factor in the potential for enhancement of ZIKV infection by Dengue antibodies that has been demonstrated *in vitro*^37^,^38^. Also definitive diagnosis of ZIKV infections will be difficult in the background of pre-existing Dengue and other flavivirus antibodies that are known to be broadly cross-reactive^39^,^40^. Such cross reactive antibodies could potentially impair the assessment of vaccine efficacy particularly in the absence of knowledge whether heterologous flavivirus antibodies can quench or potentiate immune response to the vaccine. These are major challenges to be addressed during further pre-clinical and clinical development of the vaccine.

Inactivated vaccines are easily administered, have good safety profile and might be preferable for vaccinating women of child bearing age in case of an emergency during an epidemic outbreak. Our study has demonstrated that the choice of ZIKV strain may not be a limiting factor in vaccine development. This study is a pioneering effort in ZIKV vaccine space that was initiated in late 2014, and was limited by the choice of virus strain at that period. In the absence of strain specific differences in neutralizing potential, the purified MR 766 virus strain with its ability to grow to high titers in Vero cells might pave the way for development of an effective vaccine. Availability of vaccine is important as an emergency response in regions with impending threat of ZIKV epidemics, and in the long term to restrict local transmission in regions endemic for the virus.

## Methods

### Cell and ZIKV culture.

Vero cells were derived from GMP cell banks that were prepared and extensively characterized at BioReliance, USA. The *Aedes albopictus* C6/36 cells were obtained from National Centre for Cell Sciences (NCCS), Pune and certified at the source laboratory. The C6/36 cells were tested free of mycoplasma prior to use. The cells were maintained at 27 °C in Eagle’s minimum essential medium (MEM) supplemented with 10% fetal bovine serum (Moregate Biotech, Australia). The Zika virus strain MR 766 was purchased from ATCC (ATCC, VR-84). The virus was passaged twice in C6/36 cells and was used to infect Vero cells in 6-well tissue culture plates for plaque purification using an agarose overlay. After two rounds of plaque purification, virus from a single well isolated plaque was amplified once in Vero cells. Vero cell supernatant was harvested at 80–90% CPE (cytopathic effect) and clarified by centrifugation. The virus stocks of MR 766 used in animal challenge studies were stored with 10% (v/v) fetal bovine serum in aliquots at −80 °C. The virus titer was 10^7.40^ plaque forming unit (PFU)/ml. The identity of Zika virus was confirmed by genomic RNA sequencing by Next Generation Sequencing (NGS) platform using Illumina NextSeq500 (Genotypic Technology Pvt. Ltd, Bangalore, India). The alignment of E protein sequences was performed with Clustal Omega (http://www.ebi.ac.uk/Tools/msa/clustalo/).

### ZIKV vaccine

Purified inactivated ZIKV vaccine was produced at the virus vaccine pilot facility at Bharat Biotech, Hyderabad. Virus inoculation in Vero cells was carried out at a standardized MOI of 0.01 PFU/cell and the virus was harvested at 5–6 days post-inoculation. The virus harvest was clarified with 0.45 micron filter using a tangential flow filtration (TFF) system and purified on Capto^TM^ Core 700 (GE Healthcare Life Sciences, Pittsburg, USA) column, concentrated by TFF and inactivated with 0.04% of formalin for 7 days at 22 °C in the presence of added stabilizers. Formalin was removed by diafiltration. Residual formalin content in the inactivated vaccine was estimated with acetylacetone reagent as described in the monograph 2.3.20, IP, 7^th^ edition, p99, 2014 and was below the specified limit of 0.01% w/v (WHO, TRS 963, Annexe 1, 2011). Inactivation was considered as complete when no infectious particle could be detected by plaque assay after three serial amplifications of the sample (1:3 diluted with DMEM) *in vitro* in Vero cells for 7 days. The purified inactivated ZIKV antigen was formulated with 0.25 mg aluminum per dose using aluminum hydroxide (2% Alhydrogel, Brenntag Biosector, Denmark).

### Efficacy in AG129 mice

Vaccine testing in AG129 mice and virus challenge with ZIKV strains MR 766 and FSS 13025 was outsourced to IBT Bioservices, Gaithersburg, MD, USA. All the study protocols were approved and carried out in accordance with the guidelines set by the Noble Life Sciences (NLS) Animal Care and Use Committee. Veterinary care was in accordance with the guidelines of the Public Health Service Policy, U.S. Dept. of Agriculture (USDA) and AAALAC (Association for Assessment and Accreditation of Laboratory Animal Care) International requirements. Animals were randomly allocated to groups. The study tested the efficacy of two doses of alum adsorbed 10 μg vaccine in 4–6 week old female AG129 mice (*n * = 8/group) against challenge with ZIKV. The groups 1 and 3 were vaccinated on day 0 and day 21, and groups 2 and 4 received equivalent volume of placebo (alum only) by intramuscular route (100 μl total, 50 μl in each hind leg). Prior to virus challenge, all the mice were microchipped for daily temperature monitoring. The mice in groups 1 and 2 were challenged with 10^4^ PFU of FSS 13025 via subcutaneous route on day 28. The mice in groups 3 and 4 were challenged with 10^4^ PFU of MR766 subcutaneously on day 28. Three mice from each group were test bled for serum by retro-orbital route on days 4 and 6 post infection, and were assessed for viremia by plaque assay. All the groups were monitored for weight loss, clinical score, mortality, and body temperature daily for 20 days post-challenge or until time of sacrifice. Mice displaying severe illness as determined by >20% weight loss, a health score of 5 or above (see Table 1), extreme lethargy, and/or paralysis were euthanized in accordance with the 2013 American Veterinary Medical Association (AVMA) Guidelines on Euthanasia.

### Immunogenicity in Balb/c mice

The experiments in Balb/c mice were carried out at the animal facility in Bharat Biotech, Hyderabad, India. All the animal studies and the experimental protocols were carried out in accordance with the guidelines of the Committee for the Purpose of Control and Supervision of Experiments on Animals (CPCSEA), Animal Welfare Division of the Ministry of Environment, Forest and Climate Change, Govt. of India. All the animal experiment protocols were approved by the Institutional Animal Ethics Committee of Bharat Biotech. Balb/c female mice at 6–8 weeks of age were purchased from RCC Laboratories India Private Ltd. Hyderabad, India. The animals were allocated randomly to different groups. The vaccine doses of 5 μg and 10 μg for confirmatory studies in Balb/c mice (*n* = 8/group) were rationalized from a pilot dose ranging study. The vaccine doses or alum alone (placebo) was administered intramuscularly in a volume of 100 μl (50 μl in two hind legs) on day 0 and day 21, and challenged on day 28 with 10^5^ PFU of MR 766 by intravenous route. Viremia was estimated by plaque assay every 24 hours up to 144 hours and expressed as PFU/ml. The test samples were blinded for animal injections. The samples for assays of virus and antibody titers were not blinded, but the raw data from the dose groups was blinded for independent analysis.

### Passive immunization

Two female New Zealand White rabbits of approximately 2 Kg body weight were vaccinated with two 10 μg doses of alum adsorbed purified inactivated ZIKV vaccine on days 0 and 21, and bled one week after the boost dose to obtain sufficient antisera (pooled sera log PRNT50 titer = 4.28) for passive immunization studies in mice. Four groups of Balb/c mice (*n* = 5/group) received 0.30 ml of either 1:1, 1:2, 1:4 or 1:8 dilutions (groups 1–IV) of rabbit vaccine antisera diluted with PBS, pH 7.40 by intraperitoneal route, and the control group (*n* = 5) received equivalent volume of pre-immune rabbit sera. All the groups were challenged 6 hours later with 10^5.5^ PFU of MR 766 by i.v. route. Virus titers and serum neutralizing antibody titers were estimated at 24 hours in all the groups, and the virus titer in plasma was estimated by plaque assay every 24 hours up to 144 hours post-infection, and expressed as PFU/ml. Further, plasma samples were serially amplified three times for 5–7 days *in vitro* in Vero cells for detection of infectious particles if any, by plaque assay.

### Virus titer

Plaque assays were used for virus titrations and expressed as plaque forming units (PFU/ml). Briefly, confluent Vero cells in 6-well plates were inoculated with log dilutions of the virus for 90 min in serum free MEM, followed by addition of 0.85% methyl cellulose overlay. After incubation for 4 days, cells were fixed with 10% buffered formalin, and stained with 0.1% crystal violet. Each sample was assayed in triplicates, and virus titers were calculated by multiplying the enumerated plaque count by virus dilution and volume used for infection, and expressed as PFU/ml.

### PRNT_50_

Neutralizing antibody titers were estimated by 50% Plaque Reduction Neutralization Test (PRNT_50_). Prior to the assay, 6-well tissue culture plates were seeded with 2.5 × 10^4^ Vero cells per well, and incubated at 37 °C until confluent. Four fold sera dilutions in MEM with equal volume of standardized titer of MR 766 were incubated for 90 min and added to Vero cells in the 6-well plates. After incubation for 90 min, 0.85% methyl cellulose overlay was added and incubated for 4 days. The cells were fixed with 10% formalin and stained with 0.1% crystal violet. Each sample was assayed in triplicates. Plaques were enumerated, and the estimated serum dilution causing 50% reduction in the plaques formed by the control virus sample without antibody was estimated as the PRNT_50_ titer and expressed in log values. The mean of three replicate values for each animal per time point was used in the calculation of neutralizing antibody titers by PRNT_50_. Samples with titer of ≥10 were considered as seropositive. Cross neutralization of MR 766 and heterotypic FSS 13025 strains by vaccine antisera was outsourced to IBT Bioservices, Gaithersburg, MD, USA. PRNT_50_ titers were calculated using a 4PL curve fit.

### ELISA

Binding antibody titers were estimated by ELISA. Briefly, purified inactivated ZIKV virus was coated at the standardized concentration in 96-well *Nunc*™ *MaxiSorp*™ plates overnight in 50 mM carbonate-bicarbonate buffer, pH 9.6 and blocked with 0.5% skim milk powder in coating buffer. Two fold serial dilutions of the vaccine antisera was added in triplicate wells per dilution, incubated at 37 °C for 90 min and washed with PBST (phosphate buffered saline with 0.05% tween-20) and PBS three times each. Anti-mouse-IgG HRPO secondary antibody (Sigma-Aldrich, St. Louis, USA) at 1:2500 dilution was added and incubated for 60 min, and washed three times each with PBST and PBS. Freshly prepared O-phenylenediamine dihydrochloride (Sigma Aldrich, St. Louis, USA) and hydrogen peroxide were added and incubated for 10 min. The reaction was stopped with 2 M sulfuric acid and absorbance was read at 490 nm. The mean of three replicate values was used to calculate the titer. The cut-off (endpoint titer) for seroconversion was the pre-exposure titer +3x standard deviation. The reciprocal of penultimate serum dilution above the cut-off was taken as antibody titer and expressed in log values.

### Statistical Analysis

The antibody titers were log transformed for statistical analysis using GraphPad Prism v5 (GraphPad Software, CA, USA). The values are expressed as mean with 95% c.i. Parametric paired t-tests with two-tailed limits were used to calculate differences between time points within a dose group (*n* = 8). Parametric unpaired t-test using confidence level of 95% and two-tailed limits was used to calculate variance between the groups. The values were considered statistically significant if the *P* < 0.05. The normality and homogeneity of variance assumption was checked using tests of assumptions in NCSS software version 9.0.14 prior to performing all the parametric tests.

## Additional Information

**How to cite this article:** Sumathy, K. *et al*. Protective efficacy of Zika vaccine in AG129 mouse model. *Sci. Rep.* **7**, 46375; doi: 10.1038/srep46375 (2017).

**Publisher's note:** Springer Nature remains neutral with regard to jurisdictional claims in published maps and institutional affiliations.

## Supplementary Material

Supplementary Information

## Figures and Tables

**Table 1:** Parameters for assessment of disease symptoms in AG129 mice.

Score	Abbreviation	Description	Appearance	Mobility	Attitude
1	H	healthy	smooth coat; bright eyes	active, scurrying, burrowing	alert
2	SR	slightly ruffled	slightly ruffled coat (usually only around head and neck)	active, scurrying, burrowing	alert
3	R	ruffled	ruffled coat throughout body; a ‘wet’ appearance	active, scurrying, burrowing	alert
4	S	sick	very ruffled coat; slightly closed, inset eyes	walking, but no scurrying	mildly lethargic
5	VS	very sick	very ruffled coat; closed, inset eyes	slow, or no movement; will return to upright position if put on its side	extremely lethargic
6	E	euthanize	very ruffled coat; closed, inset eyes; moribund	no movement or uncontrollable, spastic movements; will not return to upright position if put on its side	noticeable distress
7	D	deceased	—	—	—

Well-defined parameters were used to assess the severity of clinical disease following virus challenge in vaccinated and control AG129 mice. The health scores are depicted in an ascending scale of 1 to 7, with 1 for healthy, followed by progressive morbidity to death with a score of 7.
