# Supplementary material for: Protective efficacy of Zika vaccine in AG129 mouse model
Source: Sci Rep. 2017 Apr 12;7:46375. doi: 10.1038/srep46375 (PMC5388871; doi:10.1038/srep46375)
Supplement: Supplementary Information [file srep46375-s1.pdf]

Supplementary data file

Protective efficacy of Zika vaccine in AG129 mouse model

Sumathy Kandaswamy*, Bharathi Kulkarni, Ravi Kumar Gondu, Sampath Kumar Ponnuru, Nagaraju Bonguram, Rakesh Eligeti, Sindhuja Gadiyaram, Usha Praturi, Bhushan Chougule, Latha Karunakaran, Krishna M. Ella

Address:
R&D Department
Bharat Biotech International Ltd.
Genome Valley, Shamirpet
Hyderabad – 500 078, India

*Corresponding author:
Sumathy Kandaswamy, PhD
R&D Department
Bharat Biotech International Ltd.
Genome Valley, Shamirpet
Hyderabad – 500078
Tel: +91 40 27784070
sumathy@bharatbiotech.com

Supplementary Figure S1a-b.

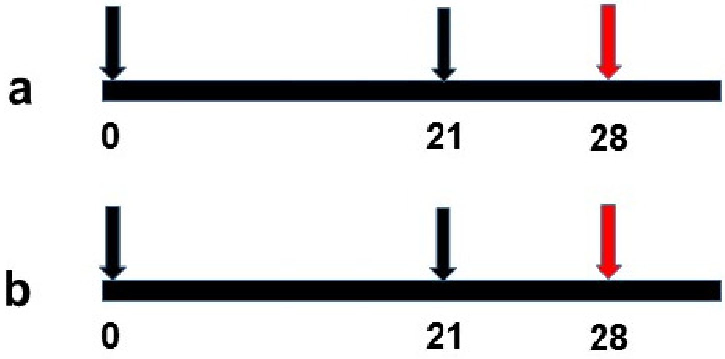

Supplementary Fig. S1a-b. Immunization and virus challenge schedule in mice. Immunization and virus challenge schedules for the ZIKV vaccine in a) AG129 mice b) Balb/c mice. Black arrows indicate vaccination, and red arrows indicate ZIKV challenge. The numbers reflect days during the study period.

Supplementary Figure S2a-b.

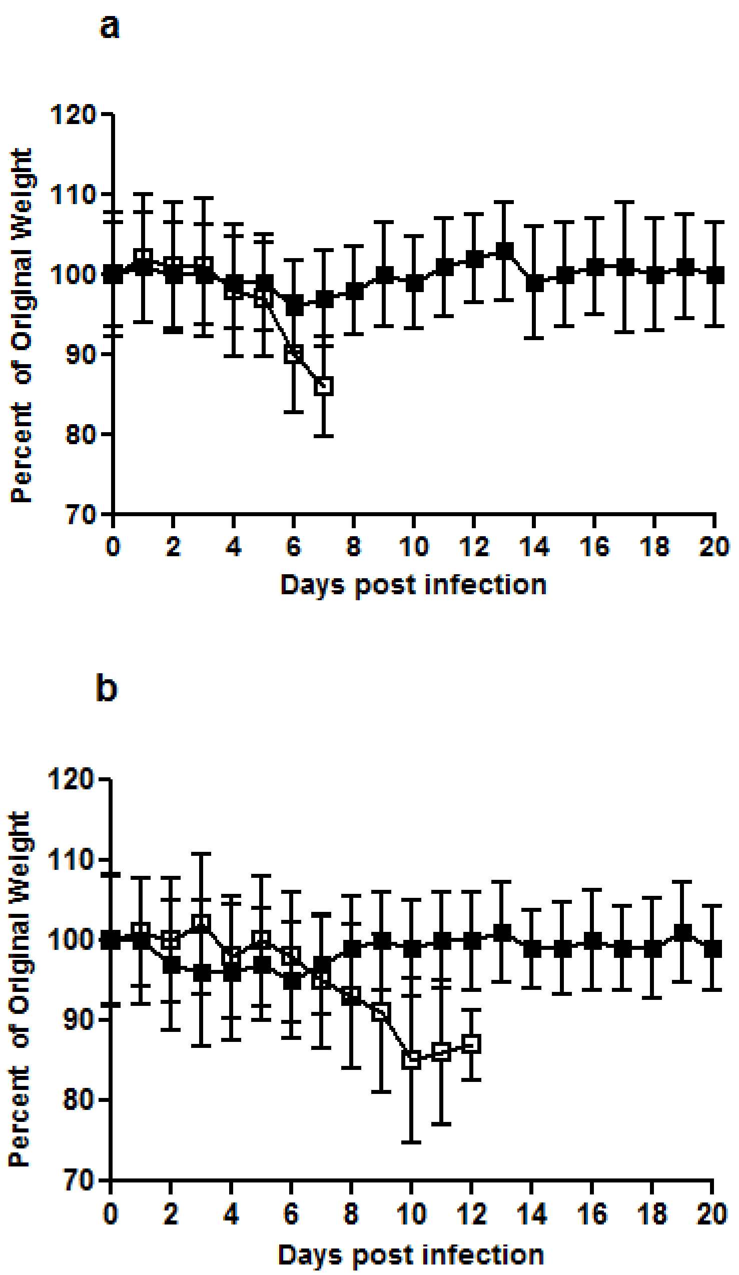

Supplementary Fig. S2a-b. Body weights following virus challenge in AG129 mice. Changes in body weight in (■) vaccinated and in (□) control AG129 mice (*n* = 8/group) following challenge with 10^4 PFU of ZIKV (a) MR 766 or (b) FSS 13025. Initial weights were assessed on individual mice on the day of virus challenge (day 0), and daily thereafter up to 20 days. The mean percent weights compared to their baseline percent weight (taken as 100%) for both the groups are plotted on day 0 as against days post-infection. Error bars represent s.d. The results are from a single experiment.

Supplementary Figure S3a-b.

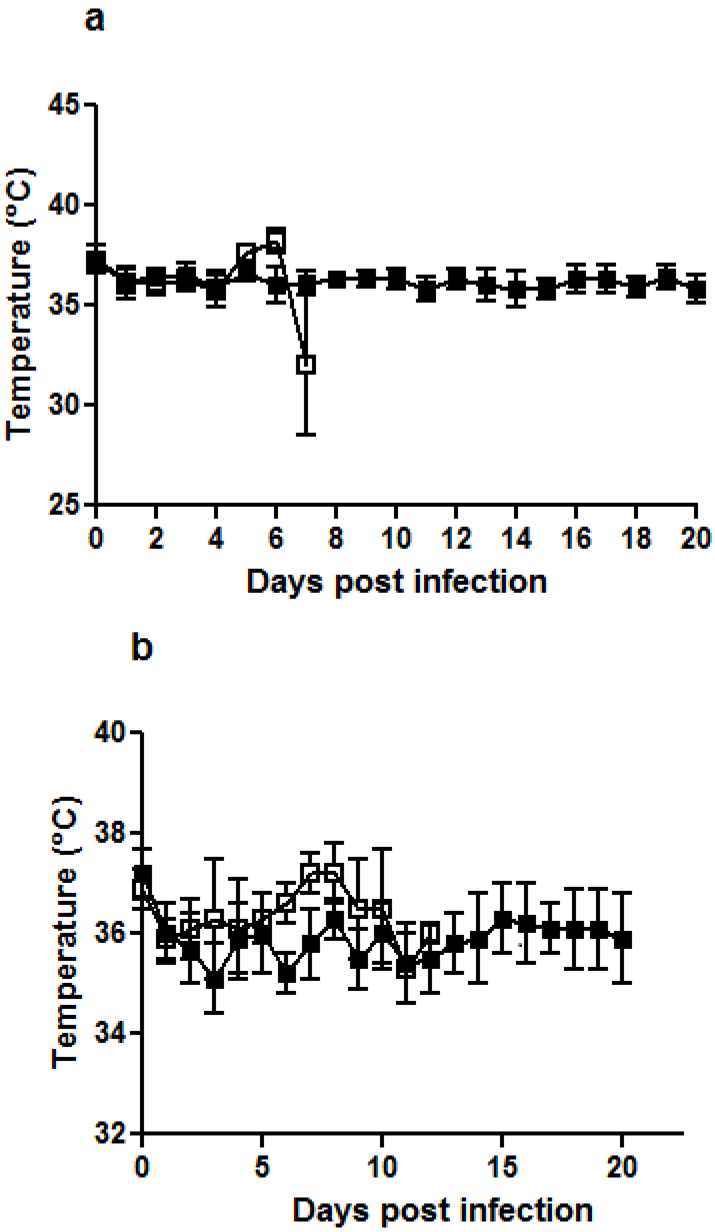

Supplementary Fig. S3a-b. Body temperatures following virus challenge in AG129 mice. Changes in body temperature in individual (■) vaccinated and in (□) control AG129 mice (*n* = 8/group) following challenge with 10^4 PFU of ZIKV (a) MR 766 or (b) FSS 13025 is expressed as mean value and plotted as against days post-infection. Error bars represent s.d.

Supplementary Figure S4a-b.

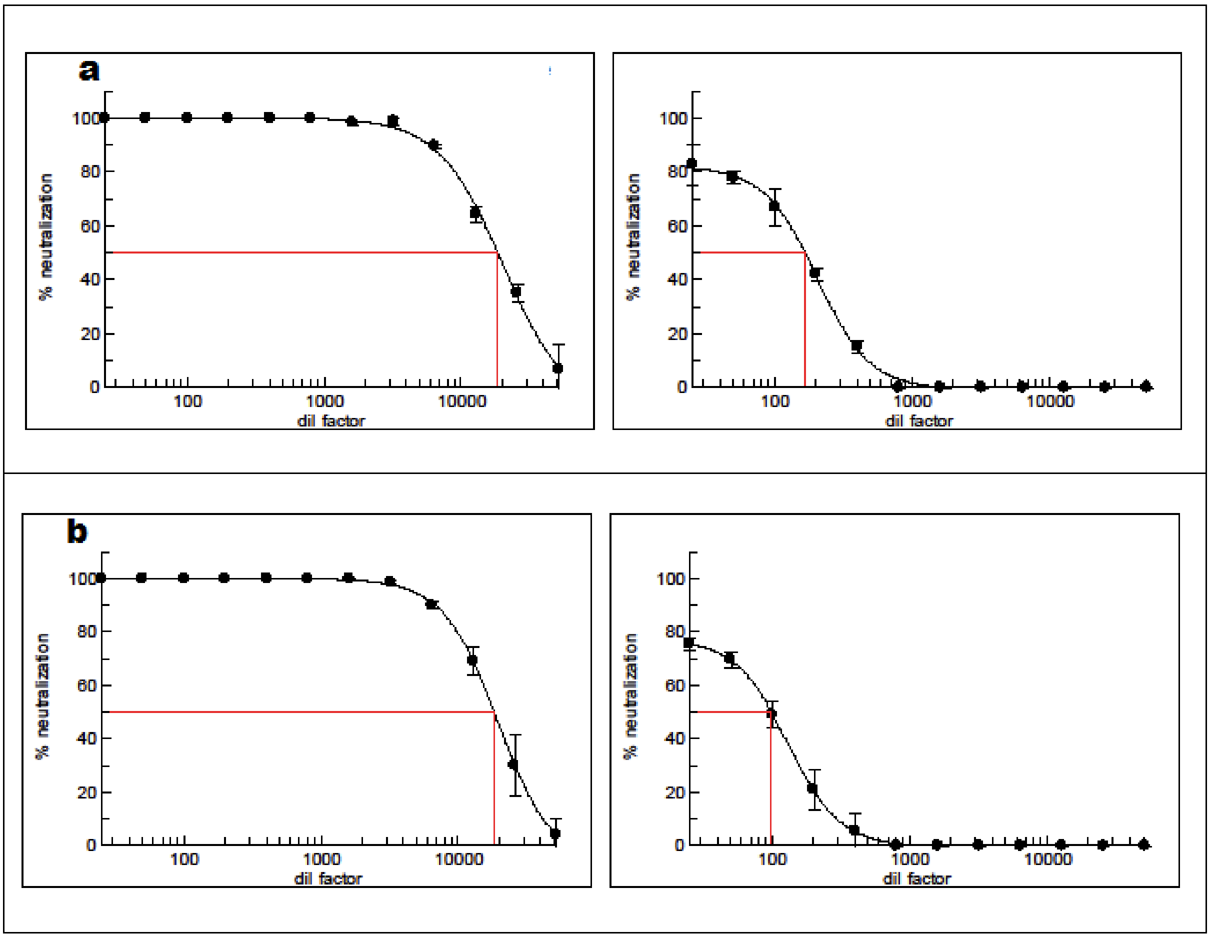

Supplementary Fig. S4a-b. Cross-neutralization of homotypic and heterotypic ZIKV strains by vaccine antisera. Vaccine antisera raised in rabbit neutralized (a) homotypic MR 766 and (b) heterotypic FSS 13025 strains with equivalent efficiency with $PRNT_{50}$ titers of 18,105 (log 4.25) and 18,325 (log 4.26) respectively. The corresponding values for placebo control sera were 165 and 99 by $PRNT_{50}$. $PRNT_{50}$ titers were calculated using a 4 PL curve fit. The titers are a mean of two replicates and the values are expressed as mean and the error bars represent s.d. Results are shown from a single experiment.

Supplementary Figure S5.

1: 1947_UGA_MR766	100.00	97.03	97.03	97.23	97.23	97.43	97.62
2: 2014_THA_KU681081	97.03	100.00	99.21	99.41	99.41	99.21	99.01
3: 2015_BRA_KU497555	97.03	99.21	100.00	99.80	99.80	99.60	99.41
4: 2013_FPN_KJ776791	97.23	99.41	99.80	100.00	100.00	99.80	99.60
5: 2016_SG_KX827309	97.23	99.41	99.80	100.00	100.00	99.80	99.60
6: 2010_CAM_KU955593	97.43	99.21	99.60	99.80	99.80	100.00	99.80
7: 2012_PHP_KU681082	97.62	99.01	99.41	99.60	99.60	99.80	100.00

Supplementary Fig. S5. Amino acid identity of the ZIKV E protein. The Clustal generated amino acid identity matrix of the E protein of ZIKV strains representative of the Asian lineage from 2010 to the more recent strain isolated in Singapore in 2016, showed a high amino acid identity of > 99.4%. The sequence id of the virus strains is represented by the year and country of isolation, followed by the GenBank Accession number. The abbreviations are UGA - Uganda, THA - Thailand, BRA – Brazil, FPN - French Polynesia, SG – Singapore, CAM – Cambodia and PHP – Philippines. The MR 766 vaccine strain and the Asian strains from 2010–2016 share > 97% identity in the E protein. The ZIKV strain FSS 13025 used in the study is 2010_CAM_KU955593.
